# The one-year incidence of stroke in patients with atrial fibrillation in Jordan and its associated factors

**DOI:** 10.3389/fmed.2024.1408249

**Published:** 2024-07-29

**Authors:** Tariq N. Al-Shatanawi, Osama Alkouri, Yousef Khader, Husam ALSalamat, Omar Aawadh Qaladi, Mohamad Jarrah, Anas Ababneh, Raed Al-Awaisheh, Taqialdeen Zamil, Ayman Hammoudeh

**Affiliations:** ^1^Department of Public Health and Community Medicine, Faculty of Medicine, Al-Balqa Applied University, Al-Salt, Jordan; ^2^Faculty of Nursing, Yarmouk University, Irbid, Jordan; ^3^Department of Community Medicine, Public Health and Family Medicine, Faculty of Medicine, Jordan University of Science and Technology, Irbid, Jordan; ^4^College of Nursing, King Saud University, Riyadh, Saudi Arabia; ^5^Division of Cardiology, Department of Internal Medicine, Faculty of Medicine, Jordan University of Science and Technology, Irbid, Jordan; ^6^Applied Science Research Center, Applied Science Private University, Amman, Jordan; ^7^Specialty Hospital, Amman, Jordan; ^8^School of Nursing, California State University, Los Angeles, CA, United States; ^9^Istishari Hospital, Amman, Jordan

**Keywords:** atrial fibrillation, metabolic abnormalities, stroke risk, diabetes, Jordan

## Abstract

**Background:**

An elevated risk of stroke is linked to atrial fibrillation (AF). Effective care and prevention measures for individuals with AF require an understanding of the factors impacting the incidence of stroke in this population. Evidence regarding the incidence of stroke among patients with AF is insufficient in Jordan. This study aimed to determine the incidence of stroke and its associated factors among patients with AF in Jordan.

**Methods:**

The Jordan Atrial Fibrillation Registry JoFib was used to identify a total of 2020 AF patients meeting the study inclusion and exclusion criteria. Demographics, clinical characteristics, and the CHA2DS2-VASc score-based evaluation of stroke risk were extracted from the registry.

**Results:**

This study encompassed 2020 participants diagnosed with AF, with 925 (45.8%) being men and 1,095 (54.2%) women. The one-year stroke incidence among the 2020 AF patients was 3.4%. Notably, stroke incidence significantly increased with age (*p* = 0.04) and was associated with the history of stroke (7.4% vs. 2.7%), hypertension (3.9% vs. 1.9%), and diabetes (5.1% vs. 2.1%). In the multivariate analysis, diabetes (OR = 2.6, 95% CI: 1.5–4.4, *p* = 0.001) and history of stroke (OR = 2.6, 95% CI: 1.5–4.6, p = 0.001) were significantly associated with stroke incidence.

**Conclusion:**

This study emphasizes Jordan’s high stroke rate among AF patients. Diabetes and prior stroke history are associated with increased odds of stroke, like all stroke patients. These results highlight the necessity for specialized management strategies among AF patients and highlight the significance of thorough risk assessment and focused interventions to reduce stroke risk in AF patients.

## Introduction

The most prevalent persistent cardiac arrhythmia, atrial fibrillation (AF), is linked to higher rates of morbidity, increased use of medical resources, and mortality. With an estimated 2–4 percent prevalence, AF affects approximately 33 million persons worldwide (1, 2). Given that a sizable percentage of patients go undetected, it is anticipated that this estimate will be underestimated (3). According to epidemiological research, more than 5.6 million Americans are likely to get AF by 2050, and by 2060, there will be 17.9 million AF patients in Europe who are older than 55 (4, 5).

A number of conditions, including coronary artery disease, heart failure, diabetes, obesity, and hypertension, are linked to an increased risk of AF (5). Likewise, these variables are linked to a higher chance of stroke and death in AF patients (3, 5, 6). Hemorrhagic strokes are caused by abrupt bleeding in the brain, while ischemic strokes are caused by obstructed blood flow to the brain. Stroke is attributed to many etiologies like atherosclerosis, cardioembolism (mainly associated with AF), small vessel disease, hypertension, aneurysm rupture, and others (7). Recent research addressed services for stroke patients in Jordan and the region with identified improvements strategies like accreditation, specialized training, and quality registries (8, 9). Patients with AF have a nearly five-fold increased risk of stroke compared to those without AF (2, 10), which accounts for around 25% of all stroke types (11). The main components of CHADS2-VASc score, which are used to assess the risk of stroke in patients with AF (12), include metabolic abnormalities linked to an elevated risk of stroke in patients with AF, the scoring items include established history of stroke/transient ischemic attack/thromboembolic event, in addition to congestive heart failure, hypertension, Age ≥ 75 years, diabetes mellitus, vascular disease (prior myocardial infarction MI, peripheral artery disease PAD, or aortic plaque), age 65 to 74 years, and sex category (female sex) (13, 14).

When compared to other forms of strokes, AF-related strokes are more deadly and cause greater death rates (11). Patients who get a stroke due to AF are twice as likely to end up bedridden as those who experience a stroke from another cause, which can be attributed to known AF before stroke (15, 16). Because of the nature of these occurrences, stroke linked to AF eventually results in a significant financial burden due to an increase in readmissions (11).

There is a lack of prior research on the incidence of stroke among patients with AF and its related variables in the Middle East, namely in Jordan. The usage of oral anticoagulants and the clinical characteristics of AF were the primary topics of the scant investigations conducted in the Middle East (17–19). Determining the incidence of stroke and the factors that contribute to it is crucial for comprehensive therapy of AF. Thus, the purpose of this study was to determine the incidence of stroke and its associated factors among patients with AF in Jordan.

## Materials and methods

### Study population

The Jordan Atrial Fibrillation (JoFIB) registry is a prospective, multicenter observational registry that, from May 2019 to January 2021, included consecutive AF patients who were over the age of eighteen in 19 hospitals and 11 outpatient clinics throughout Jordan. The methodology was already released in print (20). As an overview, data were gathered at enrollment and one, six, and 12 months following the initial examination utilizing a standardized clinical data form. A 12-lead electrocardiogram (EKG) rhythm strip lasting more than 30 s, more than one episode of AF on an ambulatory EKG monitor, or a previous diagnosis made by a treating cardiologist were used to confirm the diagnosis of AF. The usage of OACs and other pharmaceutical drugs, laboratory results, EKG, clinical and demographic profiles, and transthoracic echocardiographic features were all included in the baseline data. The classification of AF kinds, such as paroxysmal, persistent, long-standing, and permanent, and the computation of each patient’s CHA2DS2-VASc and HAS-BLED scores were done using standard definitions (12, 21). The 2019 focused update of the 2014 AHA/ACC/HRS guideline for the management of patients with atrial fibrillation was used to analyze eligibility for oral anticoagulant medicines (22).

### Inclusion criteria and baseline data collection

Patients with AF who were above 18 years old and had been diagnosed with the condition with a 12-lead EKG rhythm strip lasting more than 30 s or an ambulatory EKG monitor displaying more than one episode of AF met the inclusion criteria. Demographics (age, gender, smoking history), clinical traits body mass index [BMI], self-reported comorbidities (ischemic heart disease, hypertension, diabetes, and dyslipidemia), and the CHA2DS2-VASc score were used to estimate the risk of stroke with no cut-off value for inclusion in the study (12, 23, 24).

### Ethical considerations

The study’s participating hospitals, which included Amman Surgical Hospital, Arab Medical Center, Essra Hospital, Ibn Haitham Hospital, Islamic Hospital, Istishari Hospital, Jordan Hospital, Khalidi Medical Center, King Abdullah University Hospital, King Hussein Medical Center, Prince Hamza Hospital, Prince Hashem Hospital, Queen Alia Cardiac Center, Salt Medical Center, and Specialist Hospital, all received ethical approval (approval number 10/2021/415). Written informed permission was acquired by each subject. The study was registered with the unique identifier NCT03917992 on clinicaltrials.gov. There was no patient or public participation in the planning, execution, reporting, or distribution of this study.

### Statistical analysis

Data analysis was done with IBM SPSS version 20. Continuous variables (means and standard deviation [SD]) and categorical variables (percentages) were described via descriptive statistics. The chi-square test was used to compare the incidence of stroke among different variable categories. To identify the factors associated with stroke, binary logistic regression was conducted. A *p* < 0.05 was considered statistically significant.

## Results

### Characteristics of participants

Our study encompassed 2020 participants diagnosed with AF, with 925 (45.8%) being men and 1,095 (54.2%) women. A total of 752 (37.2%) patients were aged 70–79 years old, and 348 (17.2%) were aged 80 or older. Among participants, 280 (13.9%) were smokers and 1,136 (76.8%) had a BMI of 25 kg/m^2^ or higher. Ischemic heart disease was present in 243 (12.0%) participants, while 310 (15.4%) had a history of stroke. Utilizing the CHA2DS2VAS score, stroke risk assessment categorized 815 (40.4%) patients as high risk and 1,205 (59.6%) as low risk. Hypertension was the most common comorbidity, affecting 1,506 (74.6%) patients, followed by diabetes in 881 (43.6%) and dyslipidemia in 909 (45.0%). Detailed patient characteristics specific to Jordanian AF patients are outlined in Table 1.

**Table 1:** Demographic and clinical characteristics of patients with atrial fibrillation, *N* = 2020.

Variable	*N* (%)
Gender	
Male	925 (45.8)
Female	1,095 (54.2)
Age in years	
<50	184 (9.1)
50–59	275 (13.6)
60–69	461 (22.8)
70–79	752 (37.2)
≥80	348 (17.2)
Current smoking	
Non-smoker	1740 (86.1)
Smoker	280 (13.9)
Body Mass Index (Kg/m^2^)	
<25	436 (21.6)
25–29.9	660 (32.7)
≥30	766 (37.9)
Ischemic heart disease	243 (12.0)
History of stroke	310 (15.4)
Strok risk (CHA2DS2-VASc score)	
Low risk	1,205 (59.6)
High risk	815 (40.4)
Hypertension	1,506 (74.6)
Diabetes	881 (43.6)
Dyslipidemia	909 (45.0)

### One-year incidence of stroke among AF patients

Table 2 delineates the one-year stroke incidence among the 2020 AF patients, indicating an overall incidence of 3.4%. Notably, stroke incidence significantly increased with age (*p* = 0.04) and was associated with the history of stroke (7.4% vs. 2.7%), hypertension (3.9% vs. 1.9%), and diabetes (5.1% vs. 2.1%).

**Table 2:** One-year stroke incidence among patients with atrial fibrillation according to demographic and clinical characteristics, *N* = 2020.

	Stroke		Total, *N*	*p*-value
	No, *N* (%)	Yes, *N* (%)		
Age				0.049
<50	182 (98.9)	2 (1.1)	184	
50–59	270 (98.2)	5 (1.8)	275	
60–69	448 (97.2)	13 (2.8)	461	
70–79	717 (95.3)	35 (4.7)	752	
≥80	334 (96.0)	14 (4.0)	348	
Gender				0.555
Male	891 (96.3)	34 (3.7)	925	
Female	1,060 (96.8)	35 (3.2)	1,095	
Body mass index (Kg/m^2^)				0.796
<25	422 (96.8)	14 (3.2)	436	
25–29.9	640 (97.0)	20 (3.0)	660	
≥30	738 (96.3)	28 (3.7)	766	
Current smoking				0.106
No	1,676 (96.3)	64 (3.7)	1740	
Yes	275 (98.2)	5 (1.8)	280	
Use of anticoagulant				0.626
No use	356 (97)	11 (3.0)	367	
Use of oral anticoagulant	1,595 (96.5)	58 (3.5)	1,653	
History of stroke				<0.001
No	1,662 (97.3)	46 (2.7)	1708	
Yes	287 (92.6)	23 (7.4)	310	
Hypertension				0.034
No	504 (98.1)	10 (1.9)	514	
Yes	1,447 (96.1)	59 (3.9)	1,506	
Diabetes				<0.001
No	1,115 (97.9)	24 (2.1)	1,139	
Yes	836 (94.9)	45 (5.1)	881	
Dyslipidemia				0.087
No	1,080 (97.2)	31 (2.8)	1,111	
Yes	871 (95.8)	38 (4.2)	909	
Cancer				0.669
No	1834 (96.6)	64 (3.4)	1898	
Yes	117 (95.9)	5 (4.1)	122	
Heart failure				0.432
No	1,440 (96.8)	48 (3.2)	1,488	
Yes	511 (96.1)	21 (3.9)	532	

### Stroke incidence in relation to prescribed medications

No statistically significant associations were found between oral anticoagulant usage or antiplatelet drugs and stroke risk (*p* > 0.05). However, a noteworthy correlation (*p* < 0.001) was observed between ticagrelor usage and stroke occurrence. Additionally, correlations were found between stroke incidence and beta blockers (*p* = 0.040), amiodarone (*p* = 0.035), calcium channel blockers (CCBs) (*p* < 0.001), and statins (*p* = 0.035). No significant correlations were found with other medications. The association of stroke with prescribed medications are provided in Table 3.

**Table 3:** The one-year incidence of stroke among patients with atrial fibrillation according to prescribed medications, *N* = 2020.

	Stroke		Total, *N*	*p*-*value*
	No, *N* (%)	Yes, *N* (%)		
Oral anticoagulation				0.442
None	356 (97)	11 (3)	367	
Warfarin	628 (96.3)	24 (3.7)	652	
Dabigatran	111 (98.2)	2 (1.8)	113	
Rivaroxaban	462 (96.9)	15 (3.1)	477	
Apixaban	349 (96.1)	14 (3.9)	363	
Enoxaparin	44 (93.6)	3 (6.4)	47	
Edoxaban	1 (100)	0 (0)	1	
Antiplatelet				
Aspirin				0.209
No	1,220 (97)	38 (3)	1,258	
Yes	731 (95.9)	31 (4.1)	762	
Clopidogrel				0.711
No	1,698 (96.6)	59 (3.4)	1757	
Yes	253 (96.2)	10 (3.8)	263	
Ticagrelor				<0.001
No	1939 (96.7)	66 (3.3)	2005	
Yes	12 (80)	3 (20)	15	
Prasugrel				0.079
No	1946 (96.6)	68 (3.4)	2014	
Yes	5 (83.3)	1 (16.7)	6	
Any antiplatelet				0.208
No	1,193 (97)	37 (3)	1,230	
Yes	758 (95.9)	32 (4.1)	790	
Two antiplatelets				0.364
No	1833 (96.7)	62 (3.3)	1895	
Yes	118 (94.4)	7 (5.6)	125	
Other medications				
Beta Blocker				0.040
No	394 (98.3)	7 (1.7)	401	
Yes	1,557 (96.2)	62 (3.8)	1,619	
Amiodarone				0.035
No	1,584 (97)	49 (3)	1,633	
Yes	367 (94.8)	20 (5.2)	387	
CCBs				<0.001
No	1735 (96.4)	65 (3.6)	1800	
Yes	216 (98.2)	4 (1.8)	220	
Digoxin				0.972
No	1,643 (96.6)	58 (3.4)	1701	
Yes	308 (96.6)	11 (3.4)	319	
Flecainide, tambocor or encainide				0.248
No	1914 (96.5)	69 (3.5)	1983	
Yes	37 (100)	0 (0)	37	
RAAS				0.559
No	1,199 (96.8)	40 (3.2)	1,239	
Yes	752 (96.3)	29 (3.7)	781	
Statin				0.035
No	1,233 (97.2)	35 (2.8)	1,268	
Yes	718 (95.5)	34 (4.5)	752	
Diuretic				0.322
No	1,190 (96.9)	38 (3.1)	1,228	
Yes	761 (96.1)	31 (3.9)	792	

CCBs, Calcium channel blockers (diltiazem or verapamil), RAAS, renin-angiotensin-aldosterone system inhibitors (including Angiotensin-converting-enzyme inhibitors and Angiotensin receptor blockers).

### Factors associated with one-year stroke incidence among AF patients

Multivariate analysis (Table 4) identified risk factors for stroke in AF patients after adjusting for variables including age, BMI, hypertension, and anticoagulant use. Diabetes (OR = 2.6, 95% CI: 1.5–4.4, *p* = 0.001) and history of stroke (OR = 2.6, 95% CI: 1.5–4.6, *p* = 0.001) were significantly associated with stroke incidence.

**Table 4:** Multivariate analysis of factors associated with stroke among patients with atrial fibrillation, *N* = 2020.

Variable	OR	95% confidence interval	*p*-value
Diabetes (yes vs. no)	2.6	(1.5–4.4)	0.001
History of stroke (yes vs. no)	2.6	(1.5–4.6)	0.001

## Discussion

In the present study, relevant data of AF patients were extracted from a national clinical trial, the Jordan Atrial Fibrillation Registry, and were assessed for stroke incidence and trends regarding associated factors to stroke burden in Jordan. This study serves as a continuum of efforts to better understand AF status and its associated comorbidities, including stroke. This is the largest study reporting stroke incidence in AF patients among Jordanian population.

According to our study’s findings, 3.4% of individuals with AF had a stroke within a year. Interestingly, the likelihood of stroke in this cohort was found to be significantly increased by the presence of diabetes or a history of prior stroke. Our observed rate of stroke incidence is in line with earlier studies carried out in Jordan, which found that patients with AF had a 4.5% one-year risk of stroke/systemic embolization (17). Furthermore, our results are consistent with regional and international research that reports stroke incidence rates in people with AF ranging from 2 to 10% (25–27).

Regionally, fragmented evidence has reported incidence of stroke among AF patients (28). Stroke in AF patients was reported to be 6.4% in Qatar (29), 9.8% in Iraq (30), 10.8% in Egypt (31), 13.5% in Saudi Arabia (32), and 14.0% in Palestine (33). A study by Zubaid. et al. (34) reported 9.0% stroke prevalence among AF patients at an extensive survey of 23 hospitals in six middle-east gulf countries (Bahrain, Kuwait, Qatar, Oman, United Arab Emirates, and Yemen).

It is in line with previous research to identify diabetes as a major risk factor for stroke in patients with AF. Diabetes mellitus is known to raise the risk of thromboembolism by a number of pathological processes, such as impaired fibrinolysis, hypercoagulability, and endothelial dysfunction (35). Patients diagnosed with diabetes had significantly higher hazard ratios for both ischemic and hemorrhagic strokes, according to research from the Emerging Risk Factors Collaboration (35, 36). As a result, it is especially important to manage AF patients who also have diabetes, with anticoagulant therapy being essential in preventing strokes, according to new guidelines from the American Heart Association (24). Furthermore, concomitant conditions such hypertension, diabetes, congestive heart failure, dyslipidemia, coronary heart disease, sleep apnea, tobacco use, and obesity that are linked to an elevated risk of AF have also been linked to an increased risk of stroke (37–40). These systemic vascular risk factors lead to atrial cardiomyopathy, which can induce AF and thromboembolism. Following the beginning of AF, the atrial contractile function and the underlying atrial cardiomyopathy deteriorate, raising the risk of thromboembolism and providing an explanation for the rise in stroke risk (41).

Despite the scope of our study, it’s worth mentioning other perspectives for the mechanism attributing the cause of AF to stroke itself (42). Associations between abnormal autonomic innervation and AF have been established, with insults to the central nervous system, such as stroke, believed to play a significant role in AF’s pathogenesis (43). AF is diagnosed in about 7% of acute ischemic stroke patients within the first 3–5 days post-stroke, increasing to 25% with prolonged monitoring, a condition termed AF diagnosed after stroke (AFDAS) (16, 44, 45). AFDAS has been classified into ECG-detected AF and AF detected on a prolonged cardiac monitor (PCM-detected AF), with ECG-detected AF associated with a higher risk of recurrent ischemic stroke (46). Several mechanisms, including cardiac autonomic nervous system imbalances and stroke location within the brain, have been proposed for AF development post-stroke (47, 48). Additionally, the ‘catecholamine surge hypothesis’ and Stroke-Heart syndrome have been identified as potential contributors (44). Nevertheless, until further evidence is available, patients with AFDAS should receive anticoagulation as per current clinical practice.

Our results highlight the significance of a comprehensive care strategy for patients with AF, encompassing the timely identification, evaluation, and treatment of coexisting metabolic disorders like diabetes to reduce the risk of stroke and avoid unfavorable health consequences. It may be possible to improve the overall cardiovascular prognosis of AF patients with diabetes by teaching them about the significance of medication adherence, dietary changes, and timely medical treatment (49, 50).

Likewise, a history of stroke was found to be an additional independent risk factor that was substantially linked to an elevated risk of stroke in individuals with AF. People who have had a prior stroke are known to have a higher chance of having another one; recurrence rates range from about 30 to 43% (51–53). One of the strongest independent risk factors linked to the occurrence of strokes was a prior history of stroke, which is consistent with earlier studies conducted in Jordan (17).

These results emphasize how important it is to implement focused interventions to improve stroke preventive methods in AF patients who have previously experienced a stroke. In this high-risk subgroup, improving adherence to anticoagulant therapy and lifestyle adjustments ought to be prioritized. Furthermore, it is critical to improve public understanding and awareness of stroke risk factors and management, especially in areas like Jordan where the stroke burden is still high (54–58). Raising awareness and gaining information can help identify stroke symptoms early and get patients access to timely care, which will ultimately improve patient outcomes and lessen the burden of stroke-related morbidity and mortality in the general community.

Our study is strong in two areas. It is the first study of its kind on AF conducted in the Middle East today. The majority of earlier research on AF was carried out five to 10 years ago. Second, the study is the first multi-center investigation of the warfarin population in the area.

To ensure transparency and scientific validity, this study does come with limitations. Observational studies may introduce bias which is mainly sampling bias. Not every research subject has been enrolled in the study in order. Additionally, while other patients in the nation may be managed by their family medicine physicians, internists, or general physicians, all study participants had their AF managed by cardiologists at health facilities. Moreover, extensive associations related to variations in exact treatment regimens with incidence of stroke among AF patients cannot be concluded. Also, the study did not specify sub-types of stroke nor the types of AF and included all types of both conditions in the final analysis.

## Conclusion

In line with earlier studies conducted in the area, the 1-year stroke incidence among patients with AF in Jordan was determined to be roughly 3%. Aligned with their influence on stroke population, our research highlights the importance of diabetes and prior stroke as important risk factors for stroke incidence in patients with AF. Nevertheless, more long-term studies are necessary to fully stratify the risk of diabetes, prior strokes, and their corresponding correlations with stroke in AF patients. Further research may also examine the possible influence of therapies aimed at these risk factors on reducing the incidence of stroke and enhancing the prognosis of individuals with AF. Improved knowledge of these correlations may help develop specialized treatment plans meant to lessen the impact of stroke morbidity and mortality among individuals with AF.

## Data availability statement

Data supporting the findings of this research are available upon reasonable request from the corresponding author.

## Ethics statement

The study was conducted in accordance with the Declaration of Helsinki. The study was approved by the Institutional Review Board of the following participating healthcare settings: King Abdullah University Hospital, Amman Surgical Hospital, Arab Medical Center, Essra Hospital, Salt Medical Center, Islamic Hospital, Jordan Hospital, Khalidi Medical Center, King Hussein Medical Center, Prince Hamza Hospital, Istishari Hospital, Prince Hashem Hospital, Queen Alia Cardiac Center, Specialist Hospital, and Ibn Haitham Hospital. The participants provided their written informed consent to participate in this study.

## Author contributions

TA-S: Conceptualization, Formal analysis, Methodology, Project administration, Resources, Software, Supervision, Writing – original draft, Writing – review & editing. OA: Data curation, Formal analysis, Methodology, Resources, Software, Writing – original draft, Writing – review & editing. YK: Conceptualization, Data curation, Formal analysis, Methodology, Resources, Validation, Writing – original draft, Writing – review & editing. HA: Conceptualization, Data curation, Formal analysis, Methodology, Project administration, Resources, Software, Supervision, Validation, Writing – original draft, Writing – review & editing. OQ: Methodology, Resources, Validation, Writing – original draft, Writing – review & editing. MJ: Methodology, Resources, Validation, Writing – original draft, Writing – review & editing. AA: Methodology, Resources, Validation, Writing – original draft, Writing – review & editing. RA-A: Investigation, Methodology, Resources, Validation, Writing – original draft, Writing – review & editing. TZ: Methodology, Resources, Validation, Writing – original draft, Writing – review & editing. AH: Conceptualization, Data curation, Investigation, Methodology, Project administration, Resources, Validation, Writing – original draft, Writing – review & editing.
